# Temporal analysis of skeletal muscle remodeling post hindlimb ischemia reveals intricate autophagy regulation

**DOI:** 10.1152/ajpcell.00174.2022

**Published:** 2022-10-17

**Authors:** Mattia Scalabrin, Viktor Engman, Amanda Maccannell, Annabel Critchlow, Lee D. Roberts, Nadira Yuldasheva, T. Scott Bowen

**Affiliations:** ^1^School of Biomedical Science, Faculty of Biological Sciences, University of Leeds, Leeds, United Kingdom; ^2^Leeds Institute of Cardiovascular and Metabolic Medicine, University of Leeds, Leeds, United Kingdom

**Keywords:** autophagy, ischemia, sestrins, skeletal muscle

## Abstract

Hind limb ischemia (HLI) is the most severe form of peripheral arterial disease, associated with a substantial reduction of limb blood flow that impairs skeletal muscle homeostasis to promote functional disability. The molecular regulators of HLI-induced muscle perturbations remain poorly defined. This study investigated whether changes in the molecular catabolic-autophagy signaling network were linked to temporal remodeling of skeletal muscle in HLI. HLI was induced in mice via hindlimb ischemia (femoral artery ligation) and confirmed by Doppler echocardiography. Experiments were terminated at time points defined as early- (7 days; *n* = 5) or late- (28 days; *n* = 5) stage HLI. Ischemic and nonischemic (contralateral) limb muscles were compared. Ischemic versus nonischemic muscles demonstrated overt remodeling at early-HLI but normalized at late-HLI. Early-onset fiber atrophy was associated with excessive autophagy signaling in ischemic muscle; protein expression increased for Beclin-1, LC3, and p62 (*P* < 0.05) but proteasome-dependent markers were reduced (*P* < 0.05). Mitophagy signaling increased in early-stage HLI that aligned with an early and sustained loss of mitochondrial content (*P* < 0.05). Upstream autophagy regulators, Sestrins, showed divergent responses during early-stage HLI (Sestrin2 increased while Sestrin1 decreased; *P* < 0.05) in parallel to increased AMP-activated protein kinase (AMPK) phosphorylation (*P* < 0.05) and lower antioxidant enzyme expression. No changes were found in markers for mechanistic target of rapamycin complex 1 signaling. These data indicate that early activation of the sestrin-AMPK signaling axis may regulate autophagy to stimulate rapid and overt muscle atrophy in HLI, which is normalized within weeks and accompanied by recovery of muscle mass. A complex interplay between Sestrins to regulate autophagy signaling during early-to-late muscle remodeling in HLI is likely.

## Graphical abstract

**Figure:**
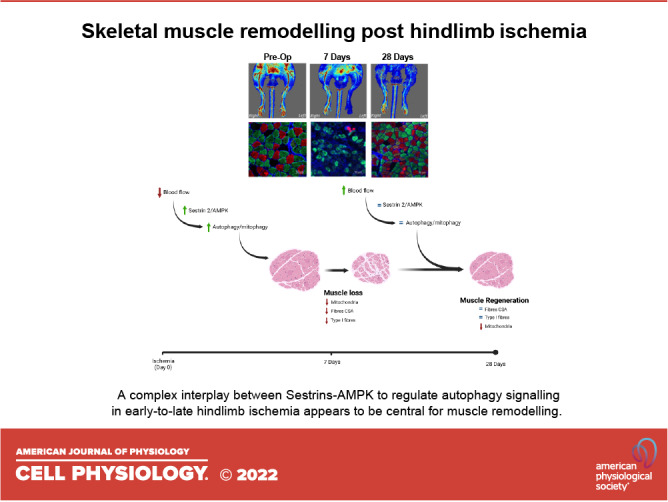

## INTRODUCTION

Hind Limb Ischemia (HLI) is the most severe form of peripheral vascular disease in humans, affecting over 200 million people worldwide (1–3). HLI reduces lower limb blood flow to cause symptoms of pain and disability, with limb amputation and death also reported (2, 3). A major outcome in patients with HLI is severely reduced functional mobility, which worsens within 6 mo of diagnosis (4, 5). The underlying mechanisms that contribute to functional decline in patients with HLI are poorly established. Impairments to skeletal muscle homeostasis are strongly implicated, which may include changes related to loss of innervation (6), fiber atrophy, contractile dysfunction, increased ectopic fat deposition, and mitochondrial derangements (4). However, there remains a paucity of data explaining what molecular events contribute toward this temporal and functional decline in muscle subjected to HLI.

HLI is associated with early-onset muscle wasting, which is closely linked with functional disability (7). Muscle mass is controlled by a complex interplay between anabolic and catabolic signaling pathways (8), with macroautophagy (herein referred to as autophagy) as a major catabolic component (9). Autophagy is vital for maintaining cellular homeostasis (10) given its role in delivering dysfunctional proteins and organelles to the autolysosome for degradation (11). However, perturbed regulation leading to sustained increases or decreases in autophagy results in overt muscle pathology (9). Previous studies using different models of HLI including cerebral ischemia (12), ischemia/reperfusion (13), and femoral occlusion (14–16) implicate autophagy as a central mechanism in the muscle wasting process. Noteworthily, autophagy seems to be upregulated early [i.e., within 2 h postischemic injury (15)] but evidence indicates that despite driving atrophy, this activation may promote muscle survival and revascularization (16). Hence, autophagy could be critical for normal muscle regeneration and physiological recovery in HLI.

Autophagy is regulated by a network complicated mirage of upstream signaling mechanisms (17). Among these, a family of newly discovered small stress-induced proteins called Sestrins has been suggested as potential master regulators of skeletal muscle homeostasis and autophagy (18). The Sestrin family contains three proteins (Sestrin 1–3) (19), with Sestrin 1 and Sestrin 2 being the two isoforms mainly expressed in skeletal muscle (20). Recent evidence further suggests that Sestrins, whose levels decrease in several muscle atrophy conditions and aging, promote skeletal muscle homeostasis [i.e., by regulating autophagy through AMP-activated protein kinase (AMPK) (13, 20)] and regeneration via effects on muscle stem cells (21). In addition, Sestrins either directly (via their oxidoreductase activity) or indirectly [via activation of the nuclear factor-erythroid factor 2-related factor 2 (Nrf2) signaling pathway] modulate oxidative stress in muscle and accumulation of oxidative damage (22).

Overall whether progressive muscle remodeling following HLI is linked to temporal changes in autophagy signaling is poorly defined (23–26). This study explored the autophagy signaling axis during skeletal muscle remodeling in severe ischemia. Targeting autophagy regulation may offer novel therapeutic targets for patients with HLI.

## MATERIALS AND METHODS

### Animal Procedures

Twelve-week-old C57BL/6 male mice (*n* = 10) were included in this study and provided ad libitum access to standard chow and water. Experiments were performed under UK Home Office animal guidelines (Scientific Procedures) Act 1986 and received ethical approval from the University of Leeds Animal Welfare Ethical Review Body. Mice underwent unilateral surgery to induce HLI in the left lower hindlimb, with the right limb serving as control (i.e., nonischemia). Before surgery, mice were anesthetized with a mix of isoflurane 0.2% and O_2_ and alongside received an injection of buprenorphine (analgesic; 1 mg/kg sc). The femoral artery and vein were isolated, ligated, and then fully dissected to induce ischemia while preventing collateralization, as previously detailed (24, 28, 29). After surgery, the external wound was sutured and mice were maintained in warmed cages until recovery. Mice were euthanized via cervical dislocation at 7 days (*n* = 5) and 28 days (*n* = 5), and dissected skeletal muscles were weighed and then immediately snap frozen in liquid nitrogen and stored at −80°C until further analysis, whereas the soleus was prepared for histological analysis as described in *Immunohistochemical Analysis*. The number of mice per group (*n* = 5) was based on past studies (16, 23, 24, 27), which showed they were powered to detect differences in our primary measure of muscle mass.

### Laser Doppler Flowmetry

Laser Doppler flowmetry was performed on Moor LDI2-HR (Moor Systems, UK) before, 7 days, and 28 days after surgery (23). Briefly, mice were anesthetized with a mix of isoflurane 0.2%, placed on a heated map, and kept under anesthesia throughout the entire duration of the recording. Images were collected and analyzed using MoorLDI software, Version 5.3 (Moor Systems, UK), by comparing the ischemic to nonischemic limb perfusion ratio, based on flux below the level of the inguinal ligament.

### SDS-PAGE Western Blot

The gastrocnemius (GC) muscles from the left and right limbs were ground in liquid nitrogen and the resulting powder was added to 200 µL of RIPA buffer (Merk, Darmstadt, Germany) with the addition of Pierce Protease and Phosphatase Inhibitor Mini Tablets, EDTA Free (Thermo Fisher Scientific, Waltham, MA). SDS-PAGE and immunoblotting analysis were performed as previously described (30). Ponceau red (Sigma-Aldrich Ltd, Gillingham, Dorset, UK) was used to verify the effectiveness of the transfer procedure and GADPH (Cell Signalling Technology, Danvers, MA) was used as a housekeeping protein to normalize the results. Primary antibodies were detected using recommended horseradish peroxidase-linked secondary antibodies (Cell Signalling Technology, Danvers, MA; see Supplemental Table S1), and chemiluminescent signal was detected using the G.Box imaging system (Syngene, Cambridge, UK) following addition of ECL (Thermo Fisher Scientific, Waltham, MA). Analysis of densitometry was performed using ImageJ software, as previously described (30). A representative image of the protein ladder (Thermo Fisher Scientific, Waltham, MA) used to determine the molecular weights during the experiments is presented in Supplemental Fig. S1.

### Immunohistochemical Analysis

Soleus muscles were mounted directly on a cork disk, surrounded with OCT mounting medium (Thermo Fisher Scientific, Waltham, MA), frozen rapidly in isopentane cooled in liquid nitrogen, and sectioned (12 μm) using a cryostat (Leica CM1850, Leica, Wetzlar, Germany) as previously described (31). To assess fiber cross-sectional area (CSA) and fiber type distribution, sections were rehydrated and blocked for 1 h in 5% goat serum (Thermo Fisher Scientific, Waltham, MA) + M.O.M. blocking (Vector Lab, Burlingame, CA). Sections were then incubated for 60 min with BA-D5 (IgG2B, 1:250 - MyHCI fibers) and SC-71 (IgG1,1:250 - MyHCIIa fibers) (Developmental Studies Hybridoma Bank, Iowa City, IA) and respective secondary antibodies (conjugated goat anti-mouse IgG2b, 1:500 - Thermo Fisher Scientific, Waltham, MA). Muscle fiber boundaries were labeled using wheat germ agglutinin, rhodamine (1:1,000; Vector Lab, Burlingame, CA). Slides were then imaged at magnification of ×20 using the Zeiss Axioscan Z1 slides scanner (Zeiss AG, Jena, Germany). Sections were analyzed using Myovision (University of Kentucky) and ImageJ software. To stain for fiber boundaries and nuclei localization, sections were fixed with 100% ice-cold methanol and then incubated for 10 min in wheat germ agglutinin, rhodamine (1:1,000; Vector Lab, Burlingame, CA). Slides were then mounted using a mounting media with DAPI (Vector Lab, Burlingame, CA) and visualized on a Zeiss Axioscan Z1 slides scanner (Zeiss AG, Jena, Germany).

### Citrate Synthase Assay

The citrate synthase assay was performed using a previously published protocol (32). Briefly, GC muscles were cryopulverized and resulting powder was added to 200 µL of RIPA buffer (Merk, Darmstadt, Germany) with the addition of Pierce protease and phosphatase inhibitor mini tablets, EDTA free (Thermo Fisher Scientific, Waltham, MA). Samples were sonicated three times for 15 s and centrifuged at 12,000 *g* for 10 min at 4°C. Supernatant was collected and protein content was quantified using the bicinchoninic acid assay. Citrate synthase activity was measured by detecting the transfer of sulfhydryl groups to 5,5′-dithiobis (2-nitrobenzoic acid) (DTNB) at a wavelength of 412 nm with readings performed every 20 s for a total of 6 min using a PowerWave HT plate reader (BioTek, Vermont, Canada). Before performing the assay, 1 µL of sample was added to the plate reader together with 199 µL of the reaction solution (100 mM Tris·HCl, 0.2 mM acetyl CoA, 0.1 mM DTNB; pH 8.1) and incubated for 5 min at 37°C. Following incubation, an endpoint reading of the background signal was performed before adding 10 µL of oxaloacetate (10 mM) to begin the experiment. Each sample was run in triplicate, means normalized to protein content, and calculated as µmol/min/mg; data are presented as percentage of control.

### Statistical Analysis

Statistical analysis was performed using IBM SPSS statistic version 22 software (IBM Analytics, New York). All tests were carried out with a 95% confidence interval and the level of significance was set at 0.05. Normal distribution was checked using the Shapiro–Wilk test while the Levene’s test was used to verify the equality of variance in our groups. Data were expressed as the mean ± standard error mean (SEM). Independent sample two-tailed *t* test was used to detect differences between the control and the ischemic groups unless otherwise noted. When the normality of distribution assumption was not met, the Mann–Whitney *U* test was used. Outliers were detected using the established ROUT statistical method (33) and the recommended Q (maximum desired false discovery rate) of 1%, with the final sample size for each experiment noted in each figure legend.

## RESULTS

### Limb Blood Flow and Muscle Remodeling Post Ischemia

**Figure 1.**
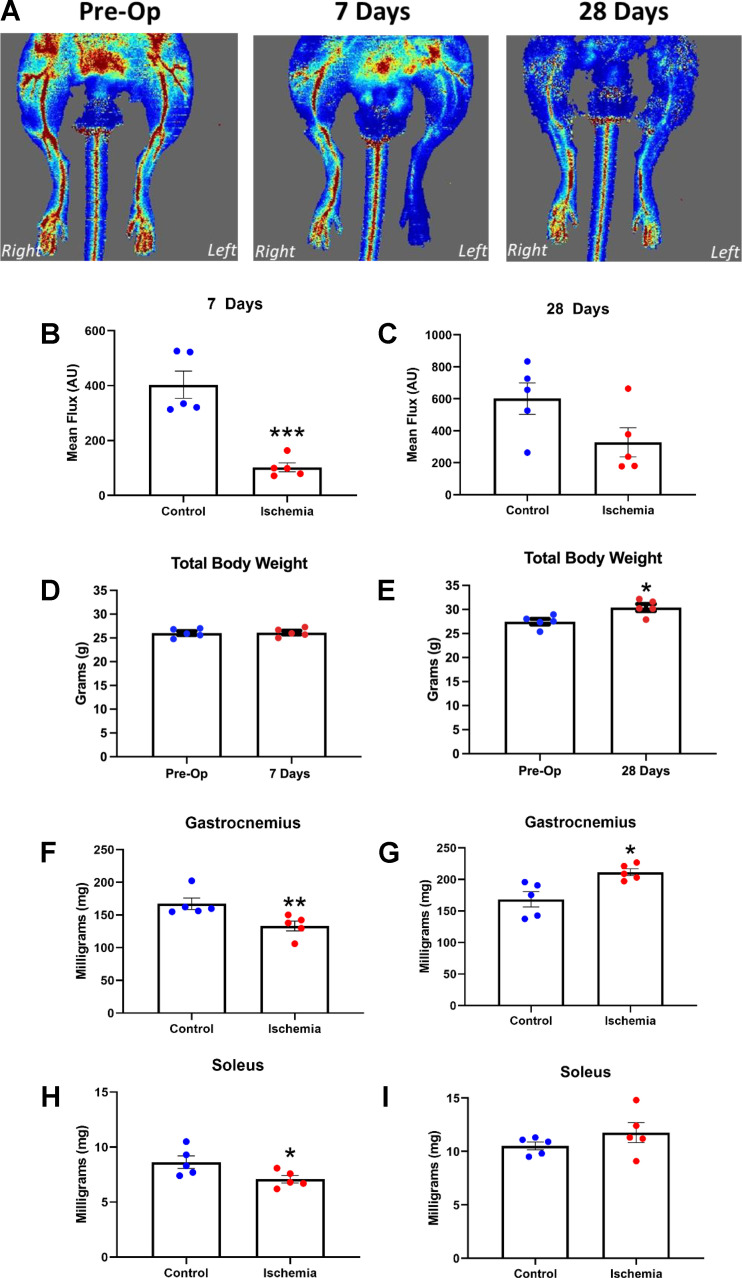
Representative images of blood flow in the lower hind limbs before (Pre-Op), 7 days, and 28 days after surgery (*A*). At 7 days post-HLI, blood flow (red/yellow) was significantly reduced in ischemic leg (−73%, *P* < 0.001; *B*) compared with contralateral limb. However, at 28 days postischemia, the blood flow was partially restored (−44%, *P* > 0.05; *C*) as a result of femoral artery collateralization. No differences in total body mass were seen 7 days post-HLI (*P* > 0.05; *D*); whereas a significant increase was seen in total body weight after surgery at 28 days (*P* < 0.05; *E*). Muscle wet weight was significantly decreased in the GC (*P* < 0.01; *F*) and soleus (*P* < 0.05; *H*) 7 days post-HLI but recovered at 28 days in both GC (*H*) and soleus (*I*). Histograms represent the mean and the standard error of the mean for each experimental group (*n* = 10). *P < 0.05, **P < 0.01, ***P < 0.001 compared with the control group. GC, gastrocnemius; HLI, hind limb ischemia.

**Figure 2.**
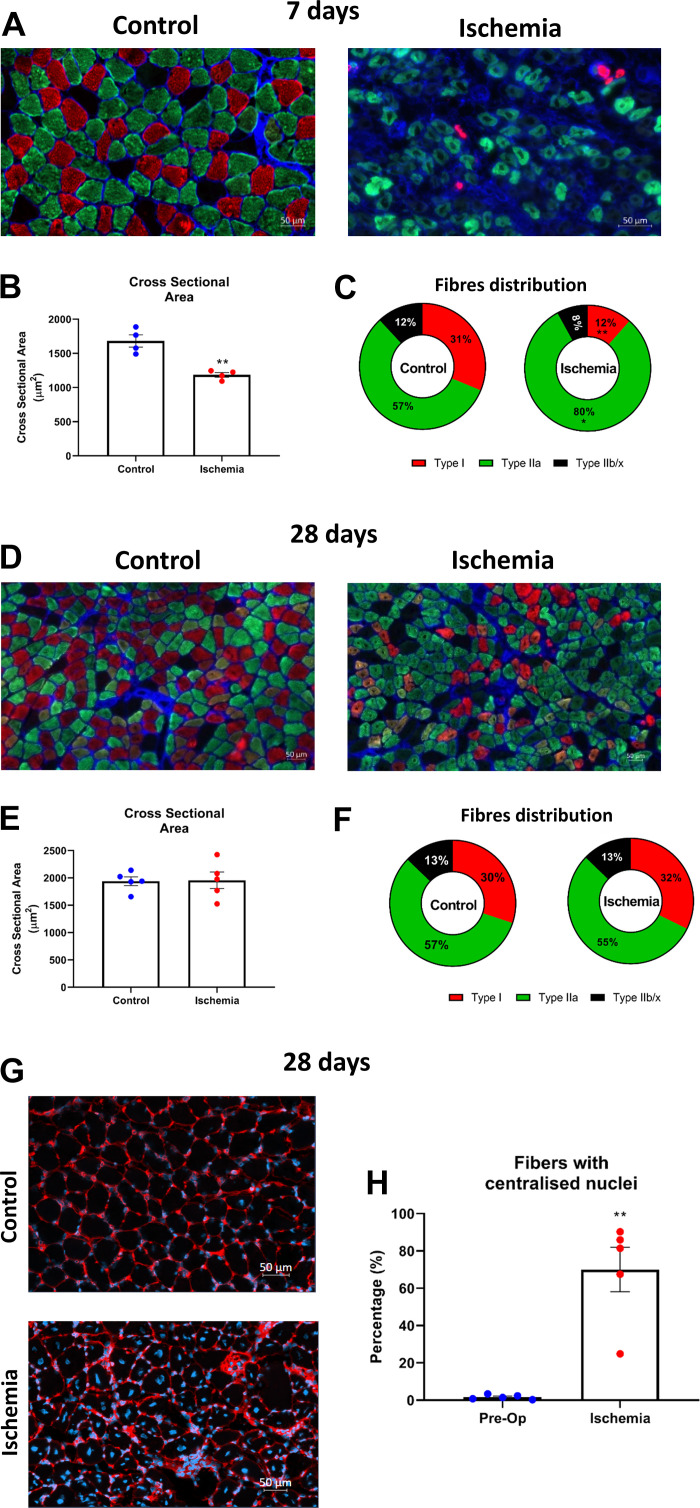
Representative images of sections from nonischemic (control) and 7 days post-HLI soleus muscle stained for MyHC isoforms (type I - red, type IIa - green, type IIb/x - black, fiber boundaries - blue) (*A* and *D*). At 7 days post-HLI, there is a significant reduction of fiber CSA (*n* = 8, *P* < 0.01; *B*) with an absolute loss of type I fibers (*n* = 8, −20%, *P* < 0.01; *C*) compared with contralateral limb. In line with the recovery of muscle mass seen at 28 days post-HLI, CSA is recovered with no differences compared with the contralateral limb (*n* = 10, *P* > 0.05; *E*) and with reappearance of type I fibers (*n* = 8, *P* > 0.05; *F*). A robust regenerative potential at 28 days was confirmed by a significant increase in fibers with centralized nuclei in the ischemic muscle compared with contralateral (*n* = 10, +68, *P* < 0.01; *G* and *H*). Histograms represent the mean and the standard error of the mean for each experimental group. *P < 0.05, **P < 0.01 compared with the control group. CSA, cross-sectional area; HLI, hind limb ischemia.

HLI was confirmed by analyzing pre- and postlimb perfusion in the control versus ligated limb (Fig. 1*A*). At 7 days postsurgery, the mean blood flux in the lower hind limb was impaired versus contralateral limb [−73%; U(8) = −2.611, *P* < 0.05; Fig. 1*B*). However, perfusion was increased at 28 days postsurgery by one-third (−44%; *P* > 0.05; Fig. 1*C*), indicative of partial revascularization of the lower hindlimb. Despite no change in total body mass (Fig. 1, *D* and *E*), 7 days of HLI resulted in decreased muscle wet-mass versus contralateral limb (both GC and soleus; *P* < 0.05; Fig. 1, *F* and *H*). Histological evidence reinforced this finding, with soleus fiber cross-sectional area showing atrophy at 7 days [*t*(8) = 2.51, *P* < 0.05; Fig. 2, *A* and *B*] alongside altered fiber composition (i.e., shift from type I to type IIa, *P* < 0.05; Fig. 2*C*). In contrast, at 28 days post-HLI, wet mass in GC muscle increased versus control [*t*(5.6) = −3.243, *P* < 0.05; Fig. 1*G*], despite no differences in soleus wet mass (Fig. 1), fiber cross-sectional area, or composition (*P* > 0.05; Fig. 2, *D–F*). Interestingly, soleus showed reappearance of type I fibers toward control levels and a robust regenerative potential at 28 days, as demonstrated by increased fibers with centralized nuclei (+68%) in ischemic muscle that was in general absent in contralateral [*t*(8) = −5.731; *P* < 0.05; Fig. 2, *G* and *H*].

### Catabolic Signaling via Autophagy Is Activated at Early HLI Stages

**Figure 3.**
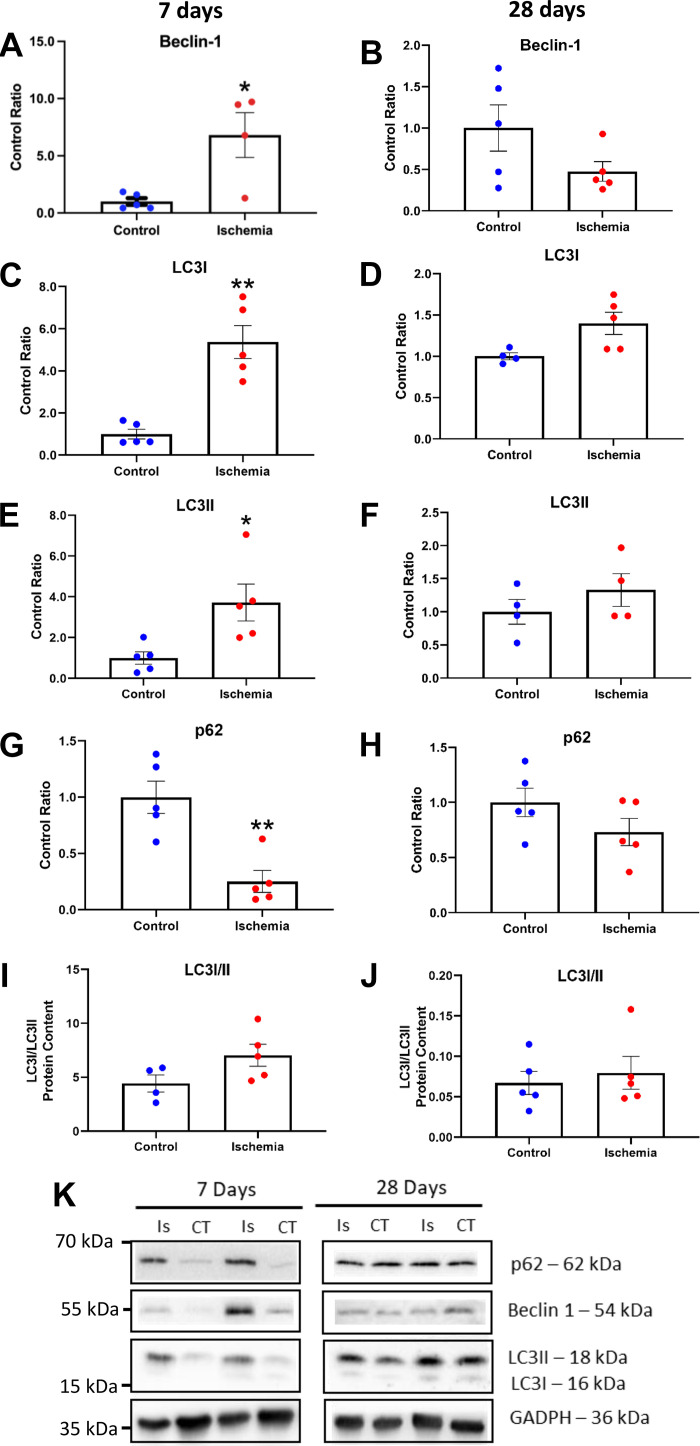
Several markers of autophagy were investigated in the gastrocnemius muscle. Beclin-1, a reliable marker of autophagy induction was significantly increased in the ischemic muscle 7 days post-HLI (*P* < 0.05, *A*). Similar trends were seen in both isoforms of LC3, a reliable marker of autophagosome formation (*P* < 0.05; *C* and *E*). Protein expression of p62, another reliable marker of autophagy which levels have been inversely correlated to autophagy activity, is instead decreased (*P* < 0.01; *G*) in ischemic muscle 7 days post-HLI reinforcing our hypothesis that autophagy is upregulated and is responsible for the loss of muscle mass seen. At 28 days post-HLI, when there is recovery of muscle mass and regeneration, the levels of Beclin-1 (*B*), LC3I (*D*), LC3II (*F*), and p62 (*H*) in the ischemic muscle are no different compared with the levels recorded in the contralateral limb. The ratio of LC3I to LC3II at 7 (*I*) and 28 (*J*) days was not different. Representative images of blots were presented (*K*). Histograms represent the mean and the standard error of the mean for each experimental group (*n* = 10). *P < 0.05, **P < 0.01 compared with the control group. HLI, hind limb ischemia; LC3, microtubule-associated protein 1A/1B-light chain 3.

Given the finding of early-onset muscle wasting after 7 days of HLI, we first explored key catabolic signaling pathways. To monitor the progression of autophagy signaling following ischemia-induced fiber atrophy, several markers were investigated in the GC muscle. Beclin-1 protein content, a reliable marker of autophagy initiation (34), was increased by sixfold versus contralateral muscle [*t*(8) = 4.943, *P* < 0.05; Fig. 3*A*) but normalized to control levels at 28 days (*P* > 0.05; Fig. 3*B*). A similar trend was found with microtubule-associated protein 1A/1B-light chain 3 (LC3), a reliable marker of autophagosome formation (34), with increased protein content of both LC3-I [*t*(8) = 14.133, *P* < 0.05; Fig. 3*C*] and LC3-II [*t*(8) = 1.965, *P* < 0.05; Fig. 3*E*] after 7 days in ischemic versus contralateral muscle with a similar trend seen in the ratio of these two proteins that did not reach statistical significance (*P* > 0.05; Fig. 3*I*). However, at 28 days, protein content of LC3-I and LC3-II and their ratio were normalized in ischemic muscle to contralateral control values (*P* > 0.05; Fig. 3*D*, *F*, and *J*). Supporting the hypothesis of increased activation of autophagy at early but not late stages of HLI, protein expression of p62 (SQSTM1) was decreased in ischemic versus control muscle at 7 days [*t*(8) = 1.632, *P* < 0.05; Fig. 3*G*) but normalized at 28 days (*P* > 0.05; Fig. 3*H*). In addition to autophagy, a major pathway mediating muscle wasting is the ubiquitin proteasome system (UPS), which is regulated in part by increased expression of key E3 ligases (termed atrogenes, i.e., MuRF1 and MAFbx). In contrast to increased autophagy signaling at 7 days, MAFbx/Atrogin-1 tended to decrease but without reaching significance [*t*(8) = 0.28, *P* > 0.05; Supplemental Fig. S1*A*], and MuRF1 protein content was decreased in ischemic muscle [*t*(8) = 3.099, *P* < 0.05; Supplemental Fig. S1*C*). At 28 days, however, atrogene expression was normalized in line with autophagy signaling (*P* > 0.05; Supplemental Fig. S1*D*). Overall, these data indicate autophagy signaling is activated at early HLI stages but with potential inhibition of proteasome-dependent catabolic activity.

### Mitophagy and Mitochondria

**Figure 4.**
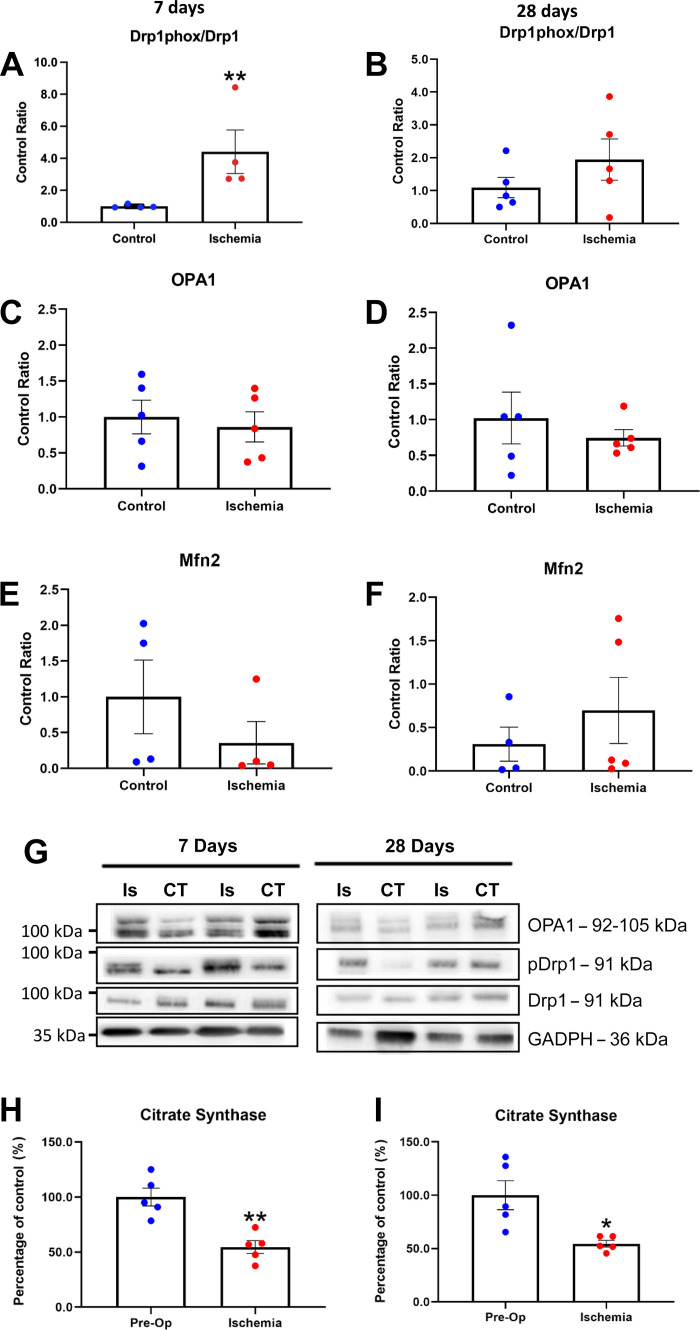
Unregulated mitophagy was seen at 7 days post-HLI with an increase of Drp1 phosphorylation (*n* = 9, *P* < 0.01) a marker of mitochondrial fission (*A*) while no changes in OPA1 content were observed (*n* = 10, *P* > 0.05; *C*). At 28 days post-HLI, the levels of Drp1 phosphorylation returned to contralateral levels (*n* = 10, *P* > 0.05; *B*) with no differences seen also in the content of OPA1 (*n* = 10, *P* > 0.05; *D*). No differences were seen in Mfn2 content at 7 (*n* = 8, *P* > 0.05; *E*) and 28 days (*n* = 10, *P* > 0.05; *F*). Representative images of blots were presented (*G*). At 7 days post-HLI, dysregulated mitophagy resulted in a loss of mitochondria measured using the citrate synthase assay (*n* = 10, *P* < 0.01; *H*), which was sustained up to 28 days post-HLI (*n* = 10, *P* < 0.05; *I*) despite the normalization of mitophagy. Histograms represent the mean and the standard error of the mean for each experimental group. *P < 0.05, **P < 0.01 compared with the control group. Drp1, dynamin-like protein 1; HLI, hind limb ischemia; Mfn2, mitofusin 2; OPA1, optic atrophy 1 protein.

An important aspect of autophagy is mitophagy, which maintains mitochondrial quality control by recycling mitochondrial proteins to preserve metabolic reserve. Mitophagy markers including phosphorylated dynamin-like protein 1 (Drp1; a marker of mitochondrial fission) increased at 7 days HLI [U(8) = −1.72, *P* < 0.05; Fig. 4*A*) despite no difference between groups for optic atrophy 1 protein (OPA-1; *P* > 0.05; Fig. 4*C*) and Mitofusin 2 (Mfn2; *P* > 0.05; Fig. 4*E*), two markers of mitochondria fusion. After 28 days, both Drp1, OPA1, and Mfn2 were not different between conditions (*P* > 0.05, Fig. 4*B*, *D*, and *F*). Given these early changes in mitophagy markers, we next measured citrate synthase activity to provide an index of mitochondrial content. Citrate synthase activity was decreased at 7 days HLI [*t*(8) = 4.568, *P* < 0.05; Fig. 4*H*) and remained reduced at 28 days compared with contralateral muscle [*t*(4.4) = 3.27, *P* < 0.05; Fig. 4*I*), which indicates an early and sustained loss of muscle mitochondrial content in HLI muscles.

### Temporal-Dependent Changes in Sestrins May Regulate Autophagy in HLI

**Figure 5.**
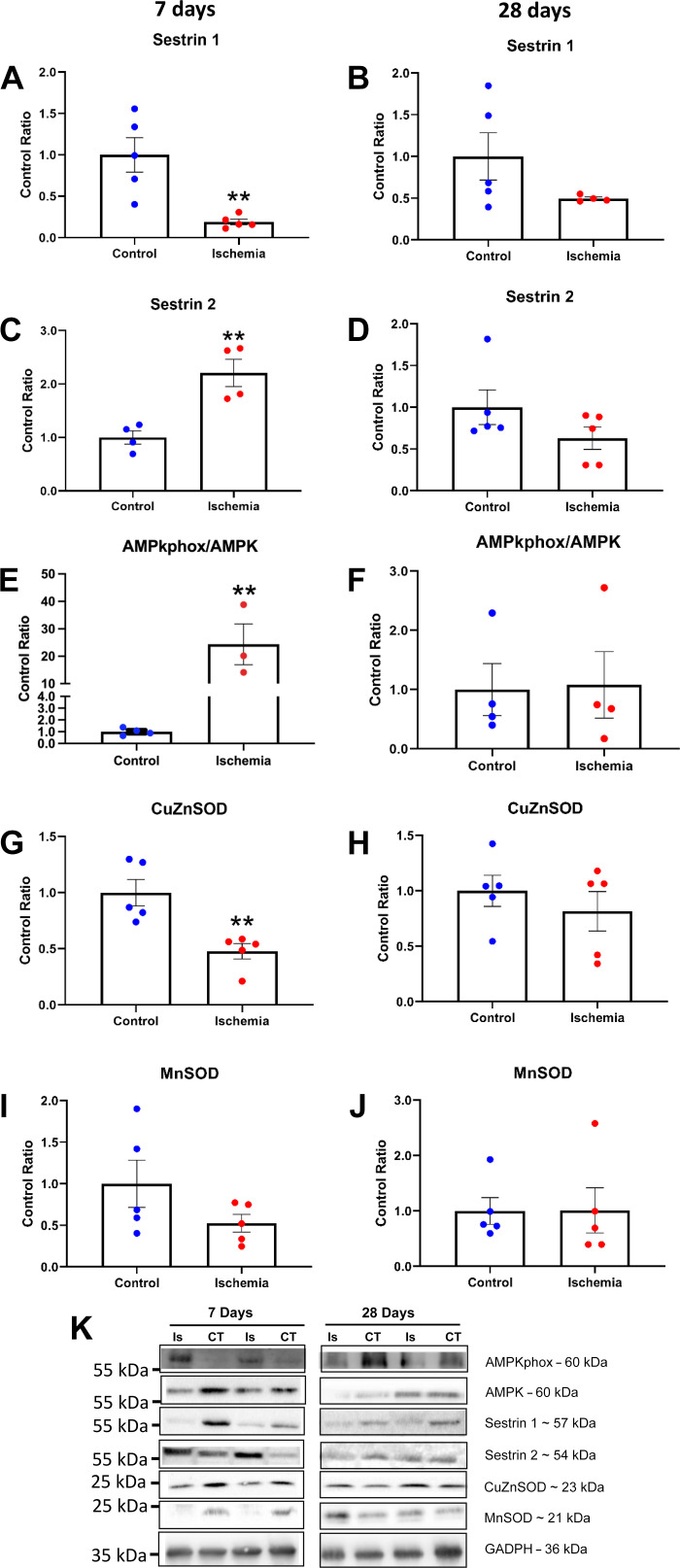
A different response was seen at 7 days post-HLI in the levels of the two subunits belonging to the Sestrin family analyzed in this study. The levels of Sestrin 1 were reduced (*n* = 10, *P* < 0.01; *A*), whereas Sestrin 2 were upregulated (*n* = 8, *P* < 0.01; *C*) suggesting a possible compensatory cross talk between the two proteins. In line with the increase of Sestrin 2 levels, the phosphorylation levels of AMPK were increased (*n* = 8, *P* < 0.01; *E*). The levels of CuZnSOD were significantly decreased 7 days post-HLI (*n* = 10, *P* < 0.01; *G*) with a similar trend seen in MnSOD (*n* = 10, *P* > 0.05; *I*). At 28-days post-HLI, the levels of Sestrin 1 (*B*), Sestrin 2 (*D*), the phosphorylation levels of AMPK (*F*), CuZnSOD (*H*), and MnSOD (*J*) returned to contralateral levels (*n* = 10, *P* > 0.05). Representative images of blots were presented (*K*). Histograms represent the mean and the standard error of the mean for each experimental group. **P < 0.01 compared with the control group. AMPK, AMP-activated protein kinase; CuZnSOD, superoxide dismutase 1; HLI, hind limb ischemia; MnSOD, superoxide dismutase 2.

Given our findings indicated that early muscle loss in HLI is associated with robust autophagy signaling alongside apparent inhibition of proteasome signaling, we next explored upstream regulators of autophagy. We first investigated whether the expression of the Sestrins family, known to influence autophagy-dependent muscle remodeling, was altered in HLI. Protein content of Sestrin 1 tended to decrease by onefold 7 days following HLI versus contralateral control muscle [*t*(4.2) = 3.832, *P* < 0.05; Fig. 5*A*]. This was in contrast to Sestrin 2, where protein expression increased by twofold [*t*(4.3) = −4.281, *P* < 0.05. Fig. 5*C*]. After 28 days of HLI, both Sestrin 1 and Sestrin 2 expressions were normalized to control values (*P* > 0.05; Fig. 5, *B* and *D*). Together, these findings suggest that a complex interplay exists between Sestrin 1 and 2 expression that could impact autophagy signaling during musclremodelingng in HLI. Sestrins regulate autophagy by modulating AMP-activated protein kinase (AMPK). AMPK phosphorylation increased in HLI at 7 days [*t*(5) = −3.755, *P* < 0.05; Fig. 5*E*) but returned to control levels at 28 days (*P* > 0.05; Fig. 5*F*).

Sestrins have inherent antioxidant properties and also regulate redox homeostasis via Nrf2. As muscle biopsies from patients with HLI show oxidative damage (4), we next assessed antioxidant expression profile. At 7 days post-HLI, expression of the mainly cytosolic antioxidant superoxide dismutase 1 (CuZnSOD) was decreased [*t*(8) = 3.827, *P* < 0.05; Fig. 5*G*], whereas the mitochondrial isoform belonging to the same family, superoxide dismutase 2 (MnSOD) tended to be reduced but without reaching significance [*t*(5.09) = 1.569, *P* = 0.177; Fig. 5*I*]. However, at 28 days, antioxidant expression was normalized to control and no differences were observed in CuZnSOD or MnSOD following HLI (*P* > 0.05; Fig. 5, *H* and *J*). Furthermore, we did not detect differences in the content of heme oxygenase 1 at both 7 and 28 days (HO-1; *P* > 0.05, Supplemental Fig. S1, *E* and *F*), an Nrf2-regulated enzyme that offers oxidative and inflammatory protection. Overall, these data suggest that HLI perturbs Sestrin signaling in line with autophagy activation and an overall downregulated antioxidant expression.

### Markers for Anabolic Signaling Were Unchanged in HLI

**Figure 6.**
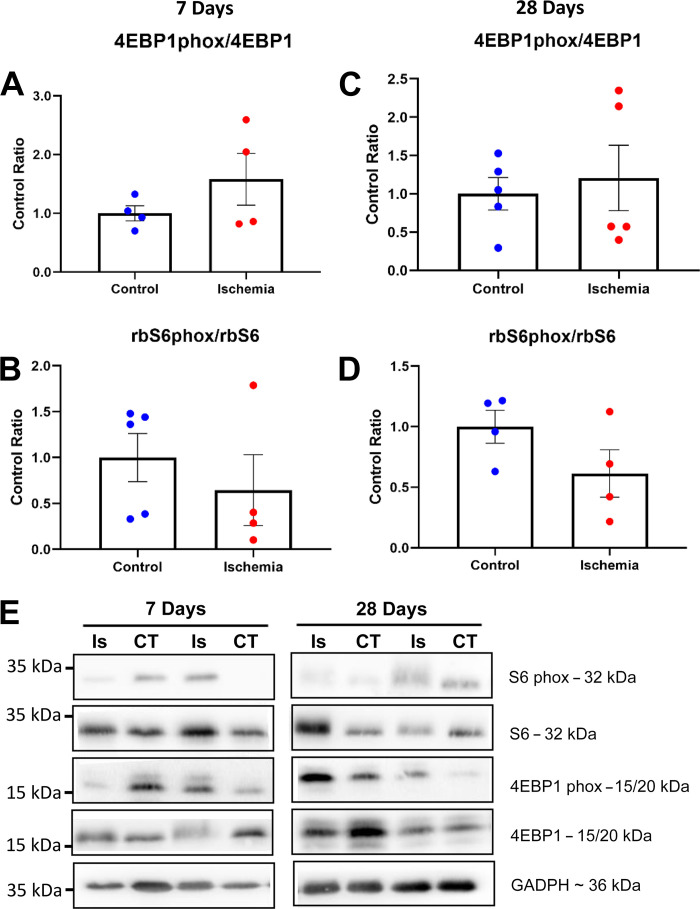
The downstream readings of the mTORC1 signaling pathway 4EBP1 (*A* and *C*) and rbS6 (*B* and *D*) were unchanged at 7 and 28 days post-HLI suggesting no activation of this signaling pathway. Representative images of blots were presented (*E*). Histograms represent the mean and the standard error of the mean for each experimental group. 4EBP1, eukaryotic translation initiation factor 4E-binding protein 1; HLI, hind limb ischemia; rbS6, ribosomal protein S6.

Given the recovery of muscle mass and cross-sectional area seen in the late-HLI group, we investigated whether HLI was affecting the regulation of protein synthesis in skeletal muscle by measuring two key readouts in the mTORC1 signaling pathway. Phosphorylation levels of eukaryotic translation initiation factor 4E-binding protein 1 (4EBP1) and ribosomal protein S6 (rbS6) remained unchanged in ischemic versus contralateral muscle both at 7 and 28 days (*P* > 0.05; Fig. 6, *A–D*).

## DISCUSSION

Our understanding of the mechanisms that cause muscle-related disability in patients with HLI remains partially resolved. In the present study, by investigating a temporal experimental model of HLI, we showed that muscle wasting occurred at early stage (7 days) but was fully normalized within weeks at late stage (28 days). Early-onset muscle wasting was closely mirrored by a robust increase in markers of catabolic-autophagy signaling. Early and sustained loss of mitochondria content in HLI was closely associated with dysregulated expression of mitophagy proteins. Our data indicate that HLI may modulate the sestrin-autophagy signaling axis to drive loss of muscle mass, given sestrin 2 was upregulated early in parallel to reduced antioxidant enzyme expression. Surprisingly, a divergent pattern for sestrin 1 expression was found, which raises the question of whether cross talk or redundancy exists in sestrins following HLI to impact muscle mass.

### Muscle Atrophy Is Associated with Activated Autophagy in HLI

Patients with HLI experience changes to muscle homeostasis that cause severe muscle wasting and disability (1, 4, 7, 23, 35, 36). A lack of consensus on the mechanisms responsible exists, however, in particular regarding the role of autophagy. In the present study, 7 days following HLI, we reported a decreased blood flow compared with the control limb (>70%), which translated to reductions in muscle mass (both GC and soleus). This muscle wasting is attributed to a 20% reduction in overall fiber CSA and an absolute loss of type I fibers following HLI, with recent suggestions that hypoxia (both environmental and pathological) may underlie such changes (37). Several different pathways are known to drive skeletal muscle atrophy and in particular the UPS and autophagy (38). The UPS is upregulated in several conditions characterized by muscle wasting (38) with MuRF1 and Atrogin-1 shown to play a pivotal role in this pathway. Our analysis showed that in ischemic muscle MuRF1 and Atrogin-1, expression is decreased when compared with the contralateral leg suggesting that during ischemia, the muscle wasting in HLI may not be driven exclusively by the UPS but rather by alternative pathways. However, it is worth mentioning that a previous study (39) reported hyperactivation of these enzymes at early stages post-HLI suggesting that, while the UPS may play an important role in the immediate aftermath of the ischemic injury, in the long-term its role may become secondary.

Several different pathways drive muscle atrophy, including autophagy (8). Autophagy is an important pathway allowing maintenance of cellular homeostasis but when dysregulated triggers muscle wasting (40, 41). Our data suggest that HLI causes early increases in muscle Beclin-1, LC3-I, and LC3-II protein content. These proteins play an important role in regulating autophagosome induction and maturation and are considered reliable markers of autophagy (42). We also found a decrease in the protein expression of p62, a cargo protein responsible for delivering dysfunctional proteins and organelles to the autolysosome for degradation being degraded itself in the process (10). Cellular content of p62 protein content is inversely correlated to autophagy (43), therefore low p62 expression reinforces our hypothesis that autophagy is upregulated in HLI and serves as a key trigger for early-onset muscle wasting. Autophagy acts to maintain a healthy pool of mitochondria in a process known as mitophagy, which can become unbalanced, thus eliminating damaged and dysfunctional mitochondria and forgoing quantity over quality (11). This is particularly relevant in HLI where muscle biopsies from patients have reduced mitochondrial number (4). At 7 days post-HLI, our data confirmed loss of mitochondria content in line with disturbed mitophagy (i.e., increased fission), as evidenced by elevated Drp1 phosphorylation (44) despite no change to markers of fusion. Mitochondria loss was sustained at 28 days following HLI, despite mitophagy markers and muscle size recovery. A disconnection between muscle mass/function and mitochondria activity does not seem to be uncommon in HLI, with a recent patient study showing that, in response to exercise, improved muscle function and fiber CSA were not associated with changes in mitochondria number and activity (45). The meaning behind the lack of recovery in mitochondrial properties compared with muscle mass remains unexplored in ischemic conditions and further studies are warranted. For example, this lag in recovery of muscle mitochondria compared with mass may explain prolonged fatigue-related symptoms experienced by patients with HLI.

### Molecular Regulators of Autophagy in HLI via AMPK and Sestrins

Several proteins are involved in maintaining a tight balance between cellular anabolism and catabolism, specifically autophagy regulation. Among these, mTORC1 plays a pivotal role in orchestrating anabolic and catabolic responses to environmental changes including autophagy inhibition (45, 46). No changes in reliable mTORC1 downstream signaling markers (4EBP1 and rbS6) were found post HLI and, despite being unable to exclude that the mTORC1 axis remains unaffected during HLI, further studies are warranted to determine levels of mTORC1 activation in HLI. To investigate other molecular regulators of autophagy, we found that phosphorylation levels of AMPK were increased following HLI at 7 days. Once phosphorylated, AMPK, a central energy sensor regulating cellular metabolism and energy homeostasis, promotes autophagy and mitophagy via several pathways. Specifically, it is known that AMPK can promote autophagy directly through the phosphorylation of specific targets in the mTOCR1, ULK1, and PIK3C3/VPS34 complexes but also by regulating transcription factors such as FOXO3, transcription factor EB, and bromodomain-containing protein 4 (47). Overall, our data suggest that upon induction of HLI, AMPK-dependent autophagy activation likely serves to accelerate muscle remodeling that exacerbates early muscle loss. Further studies are warranted to determine the specific pathway of AMPK-autophagy activation in early HLI.

Sestrins are regarded to be critical for maintenance of skeletal muscle homeostasis (20). Sestrins control autophagy to promote proteostasis that preserves muscle mass and function (18). We found Sestrin 1 content decreased but Sestrin 2 increased early following HLI. Is reduced Sestrin 1 content a compensatory response to elevated Sestrin 2 levels? Past studies have shown that in sarcopenic muscle, Sestrin 1 expression tends to decrease similar to our data in HLI (20). In contrast to Sestrin 1, Sestrin 2 is activated under hypoxic conditions induced by ischemic injury as most widely characterized by myocardial ischemia/reperfusion injury (48). Lower oxygen perfusion to ischemic muscle (15, 49, 50) may promote Sestrin 2 activation (28). Sestrin 2 regulates skeletal muscle homeostasis (48), which includes playing a pivotal role in autophagy regulation via AMPK signaling (51). It has been previously reported that Sestrin 2 can induce AMPK phosphorylation via the Serine/threonine kinase 11 to promote autophagy activation (48). By phosphorylating AMPK, Sestrin 2 appears to be a central regulator of the autophagic response following HLI. Although this may initially contribute to the wasting process, it may also be essential for supporting long-term muscle mass survival and regeneration (16, 52). For example, administration to ischemic mice of the autophagy inhibitor chloroquine reduced muscle function, regenerating potential of myocytes, despite initially conferring protection against muscle wasting (16, 52). Our data support this hypothesis, as 28 days following HLI, we found a recovery of muscle mass that was associated with normalized fiber CSA, reappearance of type I fibers, and appearance of centralized nuclei [i.e., a marker of fiber regeneration that is commonly observed during skeletal muscle repair following injury (53, 54)]. The progressive improvement of the skeletal muscle morphology, together with the metabolic changes reported, suggest that despite the initial response to HLI causing early-onset muscle wasting, this may be an important physiological response regulated by Sestrin 2 and AMPK that overall aims to protect muscle survival and enhance recovery under the most extreme stresses.

Another important role Sestrins may play in skeletal muscle is as antioxidants (i.e., directly or via regulating the NRF2 antioxidant signaling pathway) (20). Although the intrinsic catalytic activity of Sestrin 2 as an antioxidant remains unclear (48), there is evidence to suggest it promotes transcription of specific antioxidant genes including superoxide dismutase and heme-oxygenase 1 (48). Due to limited tissue availability, we were only able to measure the protein content of some antioxidant enzymes, and future studies aimed to further explore the interaction between sestrins and the NRF2 antioxidant system in HLI are warranted. Our data showed that the protein content of the antioxidant enzymes CuZnSOD and MnSOD were decreased in HLI compared with control while HO-1 remain unchanged (Supplemental Fig. S1), which aligns with other atrophic conditions characterized by oxidative stress (43, 48) and that was reported by a previous study in HLI C57Bl/6 female mice (55).

### Conclusions

In conclusion, we have shown that HLI triggers robust remodeling in skeletal muscle structure including early-onset muscle atrophy loss at 7 days that is normalized later at 28 days. Early muscle wasting following HLI muscles was associated with activated catabolic-autophagy signaling, which was closely linked to Sestrin2-AMPK signaling. Sestrins could act as potential upstream regulators of autophagy-dependent early muscle loss following HLI.

## SUPPLEMENTAL DATA

10.6084/m9.figshare.20472237 Supplemental Fig. S1 and Supplemental Table S1: https://doi.org/10.6084/m9.figshare.20472237.

## GRANTS

T.S.B. was supported by MRC UK (MR/S025472/1) and Defence And Security Accelerator (DASA - ACC2011654).

## DISCLOSURES

No conflicts of interest, financial or otherwise, are declared by the authors.

## AUTHOR CONTRIBUTIONS

M.S., L.D.R., N.Y., and T.S.B. conceived and designed research; M.S., V.E., A.M., A.C., and N.Y. performed experiments; M.S., V.E., A.M., and A.C. analyzed data; M.S., V.E., L.D.R., and T.S.B. interpreted results of experiments; M.S. prepared figures; M.S. and T.S.B. drafted manuscript; L.D.R., N.Y., and T.S.B. edited and revised manuscript; M.S., V.E., A.M., A.C., L.D.R., N.Y., and T.S.B. approved final version of manuscript.
